# Differential Accumulation and Activation of Monocyte and Dendritic Cell Subsets in Inflamed Synovial Fluid Discriminates Between Juvenile Idiopathic Arthritis and Septic Arthritis

**DOI:** 10.3389/fimmu.2020.01716

**Published:** 2020-07-31

**Authors:** Maïlys Cren, Nadège Nziza, Aurélia Carbasse, Perrine Mahe, Emilie Dufourcq-Lopez, Marion Delpont, Hugues Chevassus, Mirna Khalil, Thibault Mura, Isabelle Duroux-Richard, Florence Apparailly, Eric Jeziorski, Pascale Louis-Plence

**Affiliations:** ^1^IRMB, INSERM, Université Montpellier, Montpellier, France; ^2^Arthritis R&D, Neuilly sur Seine, France; ^3^CHU Montpellier, Pediatric Department, Université Montpellier, Montpellier, France; ^4^CHU Montpellier, IRMB, Université Montpellier, Montpellier, France; ^5^CHU Montpellier, Pediatric Orthopedic Surgery Unit, Université Montpellier, Montpellier, France; ^6^CHU Montpellier, Centre d'Investigation Clinique, Université Montpellier, Montpellier, France; ^7^Inserm, CIC1411, Montpellier, France; ^8^CHU Montpellier, Clinical Research and Epidemiology Unit, Université Montpellier, Montpellier, France; ^9^CHU Montpellier, Clinical Department for Osteoarticular Diseases, Université Montpellier, Montpellier, France; ^10^PCCI, INSERM, University of Montpellier, Montpellier, France

**Keywords:** juvenile idiopathic arthritis, septic arthritis, monocytes, dendritic cells, CD141 cDC, CD123 pDC, multiparametric flow cytometry

## Abstract

Despite their distinct etiology, several lines of evidence suggest that innate immunity plays a pivotal role in both juvenile idiopathic arthritis (JIA) and septic arthritis (SA) pathophysiology. Indeed, monocytes and dendritic cells (DC) are involved in the first line of defense against pathogens and play a critical role in initiating and orchestrating the immune response. The aim of this study was to compare the number and phenotype of monocytes and DCs in peripheral blood (PB) and synovial fluid (SF) from patients with JIA and SA to identify specific cell subsets and activation markers associated with pathophysiological mechanisms and that could be used as biomarkers to discriminate both diseases. The proportion of intermediate and non-classical monocytes in the SF and PB, respectively, were significantly higher in JIA than in SA patients. In contrast the proportion of classical monocytes and their absolute numbers were higher in the SF from SA compared with JIA patients. Higher expression of CD64 on non-classical monocyte was observed in PB from SA compared with JIA patients. In SF, higher expression of CD64 on classical and intermediate monocyte as well as higher CD163 expression on intermediate monocytes was observed in SA compared with JIA patients. Moreover, whereas the number of conventional (cDC), plasmacytoid (pDC) and inflammatory (infDC) DCs was comparable between groups in PB, the number of CD141^+^ cDCs and CD123^+^ pDCs in the SF was significantly higher in JIA than in SA patients. CD14^+^ infDCs represented the major DC subset in the SF of both groups with potent activation assessed by high expression of HLA-DR and CD86 and significant up-regulation of HLA-DR expression in SA compared with JIA patients. Finally, higher activation of SF DC subsets was monitored in SA compared with JIA with significant up-regulation of CD86 and PDL2 expression on several DC subsets. Our results show the differential accumulation and activation of innate immune cells between septic and inflammatory arthritis. They strongly indicate that the relative high numbers of CD141^+^ cDC and CD123^+^ pDCs in SF are specific for JIA while the over-activation of DC and monocyte subsets is specific for SA.

## Introduction

Septic arthritis (SA) and juvenile idiopathic arthritis (JIA) are the two most frequent arthritis types in childhood (1). SA is a bacterial infection of a joint space, mostly caused by bacteremia in pediatric cases (2). Although the main causative agent is *Staphylococcus aureus* in childhood, and *Kingella kingae* in infants and toddlers (3), the infectious agent remains however frequently undetermined. SA is an orthopedic emergency, and delay in diagnosis and treatment may result in irreversible joint damage. JIA is the most common chronic rheumatic disorder in childhood, and is classified in seven subtypes, depending on the number of affected joints, serological features, and systemic symptoms. Oligoarticular JIA is the most common subtype (4). The exact etiology and pathophysiology of JIA remain unknown, but several genetic and environmental factors have been associated with this disorder (5). Accurate and early diagnosis is critical, since SA and JIA treatments are different: surgical drainage and antibiotics for SA, and anti-inflammatory and/or immunosuppressive therapy for JIA.

Despite their distinct etiology, several lines of evidence suggest that the innate immunity plays a pivotal role in JIA and also SA pathophysiology. Dendritic cells (DCs) and monocytes are the main immune cells implicated in the innate immune response. They are involved in the first line of defense against pathogens and play a critical role in initiating and orchestrating the inflammatory response through the production of pro-inflammatory cytokines and chemokines. DCs are key players in initiating the immune response and maintaining tolerance. They sample antigens from the environment and present them as peptide-MHC complexes to effector T cells in lymphoid organs. In peripheral blood (PB), DCs are divided in three main subsets: two myeloid or conventional DCs (cDCs; BDCA1/CD1c^+^ cDCs and BDCA3/CD141^+^ cDCs), and plasmacytoid DCs (pDC) characterized by CD123 (IL-3R) expression. These three DC populations are found also in all lymphoid organs, and represent resident DCs. Previous studies showed a deficiency of blood DCs, particularly myeloid DCs and pDCs, in patients with JIA compared with healthy controls (6, 7). Furthermore, low levels of blood pDCs in JIA are associated with poor treatment response (7). Synovial fluid of inflamed joints also is enriched in myeloid DCs that strongly express maturation markers, suggesting that they might participate in the initiation and maintenance of inflammation (7). A population of synovial inflammatory DCs (CD14^+^ infDCs; CD1c^+^CD14^+^CD16^−^) has been identified in inflamed SF samples of patients with rheumatoid arthritis (RA) (8). This subset differentiates from circulating monocytes recruited to the inflammation site, and plays a role in RA pathogenesis by inducing GM-CSF, IL-17 and IFN-γ production by CD4^+^ T cells (9).

Human PB monocytes are a heterogeneous cell population that includes classical (CD14^++^CD16^−^), intermediate (CD14^++^CD16^+^), and non-classical (CD14^+^CD16^++^) monocytes. They represent ~80, 5, and 7% of all blood monocytes, respectively (10). Classical monocytes display high phagocytic activity, while non-classical cells are poorly phagocytic *in vitro*, and patrol healthy tissues through long-range crawling on the resting endothelium. This patrolling behavior is dependent on the integrins LFA-1 and ICAM1 and is required for rapid tissue invasion at the infection site in a CX3CR1-dependent manner (11). After extravasation, non-classical patrolling monocytes rapidly differentiate into macrophages, and acquire tissue repair functions (12). Classical monocytes are recruited to inflamed tissues in a CCR2-dependent manner, where they initiate the immune response and differentiate into infDCs (13). This heterogeneity suggests specialized functions. Indeed, *in vitro* activation of highly purified non-classical monocytes by stimulation with TLR-7/8 results in increased release of IL-1β and TNF-α cytokines. Conversely, purified classical monocytes respond more readily to TLR-2/4 activation by producing cytotoxic metabolites (ROS, NO) and pro-inflammatory cytokines, particularly IL-6 and IL-8 (14, 15). CD14^++^CD16^+^ intermediate monocytes exhibit an intermediate phenotype between classical and non-classical monocytes and play a major role in antigen presentation. In response to lipopolysaccharides, they mainly produce TNF-α and IL-1β. Both intermediate and non-classical monocytes are increased in PB of children with sepsis compared with healthy donors (16), and high numbers of intermediate monocytes are often observed in PB and SF of patients with autoimmune arthritis (17–20). In active arthritis, the levels of pro-inflammatory cytokines are generally higher in SF than in PB (21).

Depending on their localization and the nature of stimuli, DCs and monocytes can undergo distinct modifications of their phenotype and function. As SA and JIA have different etiology (infectious condition and auto-reactive immune response, respectively), we hypothesized that variations in the number and/or activation status of myeloid cell subtypes could help to differentiate between these conditions. Indeed, some biological parameters (e.g., synovial leukocyte count and platelet numbers) differ significantly in SA and JIA, but there is a large overlap zone and none of them allows discriminating between these conditions (22). As previous studies investigated the heterogeneity of PB and/or SF DC or monocyte subsets in JIA or SA always by comparing with healthy controls or patients with RA, but never between these rheumatic diseases, we performed an in-depth phenotypic characterization of DC and monocyte subsets in PB and SF samples of patients with SA and JIA. Our goal was to identify cell subsets known to be associated with different pathophysiological mechanisms and that could be used as disease-specific biomarkers.

## Materials and Methods

### Patients

Children, aged from 6 months to 15 years, presenting with acute arthritis requiring arthrocentesis were included if they met the diagnostic criteria for JIA or SA. Patients with JIA fulfilled the International League of Associations for Rheumatology classification criteria and were prospectively recruited from the Pediatric Department at Montpellier University Hospital. Patients with systemic JIA were excluded because joint puncture is not part of the clinical management. Patients who took steroids, biological therapy or disease-modifying anti-rheumatic drugs in the month preceding the arthrocentesis, and patients treated with antibiotics before arthrocentesis were excluded. Children with arthrocentesis contraindication (platelet count below 50,000/mm^3^, reduced prothrombin, prolonged activated partial thromboplastin time) were excluded. For patients with SA, disease was confirmed either bacteriologically after culture according to the French national guidelines, or using specific PCR assays. The study protocol was reviewed and approved by the local Ethics Committees (Comité de Protection des Personnes Sud Méditerranée I, ref 2014-A01561-46). All children/parents have been informed and a written informed consent to participate in this study was provided by the participants' legal guardian/next of kin.

### Laboratory Assays

Total white blood cells (WBC) and platelets counts, C-reactive protein (CRP) dosage, antibody analyses, and medical records were collected as part of the routine diagnostic evaluation of JIA/SA at the Department of Immunology (CHU Montpellier).

### Reagents

The V450-conjugated anti-CD3, Alexa Fluor®700-conjugated anti-CD3, BV650-conjugated anti-CD4, FITC-conjugated anti-CD4, BV605-conjugated anti-CD11b, BV421-conjugated anti-CD11c, BV650-conjugated anti-CD14, PE-Cy^TM^7-conjugated anti-CD14, Alexa Fluor®700-conjugated anti-CD16, BV421-conjugated anti-CD16, PE-CF594-conjugated anti-CD16, phycoerythrin (PE)-conjugated anti-CD19, Alexa Fluor®700-conjugated anti-CD19, Alexa Fluor®700-conjugated anti-CD20, V500-conjugated anti-CD45, BV786-conjugated anti-NKp46, Alexa Fluor®700-conjugated anti-CD56, PE-conjugated anti-CD64, PE-Cy^TM^7-conjugated anti-CD86, PerCP-Cy5.5-conjugated anti-CD123, BV711-conjugated anti-CD141, BV786-conjugated anti-PDL2, and PE-CF594-conjugated anti-CD163 antibodies were from BD Biosciences. The APC-Alexa Fluor®750-conjugated anti-HLA-DR antibody was purchased from Beckman Coulter. The PE-conjugated anti-CD1c, APC-conjugated anti-CD303, and APC-conjugated anti-SLAN antibodies were from Miltenyi Biotech. The FITC-conjugated anti-CD192 antibody was purchased from R&D Systems. All antibodies were used according to their manufacturer's recommendations. Antibodies combination for monocytes: CD3, CD11b, CD14, CD16, CD19, CD45, CD56, CD64, CD163, CD192, NKp46, HLA-DR, SLAN, and DC: CD1c, CD3, CD4, CD11b, CD11c, CD14, CD16, CD19, CD20, CD45, CD56, CD86, CD123, CD141, CD303, PDL2, HLA-DR.

### Flow Cytometric Immunophenotyping

Granulocytes, T and B lymphocytes and natural killer (NK) cells staining was performed using 50 μl of fresh, heparin-treated whole blood and antibodies against CD3, CD4, CD16, CD19, CD45, and CD56. DCs and monocytes were characterized using 300 μl of fresh, heparin-treated whole blood. After labeling with the relevant antibodies erythrocytes were gently lysed by adding the EasyLyse^TM^ solution (Dako). Synovial cell fractions were recovered by centrifugation (300 g, 10 min) of heparin-treated SF samples. One to five million cells from SF were stained using the same antibodies as for the whole blood samples after Fc receptor blocking with Fc Block (BD Biosciences).

For monocyte characterization, selection of DAPI-negative CD45^+^ viable leukocytes were performed and debris and doublets were excluded. Contaminating granulocytes (HLA-DR^neg^CD16^high^) and lineage-positive cells (CD3, CD19, and CD56) were excluded from the monocyte gate. The three monocyte populations were gated within HLA-DR-positive cells (Supplemental Figure 1). The percentage of classical (CD14^++^CD16^−^), intermediate (CD14^++^CD16^+^), and non-classical (CD14^+^CD16^++^) monocytes are given within the gated monocytes, after exclusion of double negative cells. Expression of CD64, CD163, HLA-DR, CCR2 and SLAN markers were determined on the 3 monocyte subsets from both biological fluids leading to thirty MFI parameters on monocyte. Results are expressed as delta values of the mean fluorescence intensity (MFI), calculated by subtracting negative control MFI (unlabeled cells) from MFI of the marker in the corresponding color.

For DC characterization, cells were plotted according to their size and granularity, selection of DAPI-negative CD45+ viable leukocytes and doublet exclusion were performed. Lineage-negative cells were first selected, and then CD4 and HLA-DR double-positive cells were gated to delineate the two major DC subsets: cDCs (CD11c^++^HLA-DR^++^CD123^low^CD1c^+^CD303^−^) and pDCs (CD11c^low^HLA-DR^++^CD123^high^CD303^+^) (Supplemental Figure 2). Four DC subsets were analyzed (CD1c^+^ and CD141^+^ cDCs, CD123^+^ pDCs and CD14^+^ infDCs). Expression of HLA-DR, CD86 and PDL2 were determined on the 4 DC subsets from both biological fluids leading to twenty-four MFI parameters on DC. MFI were calculated by subtracting negative control MFI from MFI of the marker in the corresponding color. BD^TM^ Cytometer Setup and Tracking (CS&T) software and BD^TM^ Cytometer Setup and Tracking beads were used to ensure consistent and reproducible results over time. Cell numeration was performed by addition of known numbers of fluorescent beads (Flow-Count^TM^ Fluorosphere, Beckman Coulter) before cell acquisition. This allowed calculating the absolute cell number for each cell subsets. All data were acquired using a BD-LSR Fortessa Cell Analyzer (BD Biosciences). Color compensation analyses were performed using the DIVA or FlowJo (Tree Star, Ashland, OR, USA) software, and all data were analyzed with FlowJo.

### Hierarchical Clustering Analyses

Percentage of monocyte subsets and all cell counts were normalized using log_2_ transformation. Delta values of MFI of cell-surface markers were normalized using an arcsinh transformation (cofactor=150). Normalized values were analyzed using the MultiExperiment Viewer (MeV) v4.10.6 software to perform non-supervised hierarchical clustering analyses. Hierarchical clustering analyses were produced using Pearson Correlation and complete linkage clustering. The two-side unpaired *t*-test was performed with a false discovery rate (FDR) cut-off of 5%. The selected markers were used to build the heatmaps.

### Statistics

For each cell subset, data are presented as the mean ± SD or ± SEM (as indicated) of the total cell counts per microliter of blood/SF and were compared using the Student's *t* or Mann–Whitney test, according to their distribution. In parallel, data were compared using multivariate linear regression models, to consider the age difference between groups and age-adjusted means were estimated for a constant age of 5.03 years. We compute partial correlation of variable with age, controlling for arthritis types, using Spearman partial correlation coefficient. The capacity of markers to discriminate between JIA and SA was assessed using the receiver operating curve (ROC) analysis with estimation of the area under the curve (AUC) values (23). As age was significantly different between patients with JIA and SA, the AUC value estimates were calculated only for parameters that remained significant after adjusted for age. A cutoff maximizing the Youden J-index (sensitivity + specificity −1) has been identified to provide sensitivity and specificity for these parameters. All the *p*-values presented, except for clinical data in Table 1, are adjusted for the FDR using the method of Benjamini (24) considering the 84 parameters analyzed (including number, proportion and MFI for each cell subsets). Analyses were performed with a bilateral alpha level of 0.05 (after FDR adjustment) using the SAS software, version 9.4 (SAS Institute, Cary, NC, USA).

**Table 1:** Characteristics of the enrolled patients.

	**JIA**	**SA**						
**Gender (F–M)**	12–3	8–6						
**ANA (pos-neg)**	11–3	NA						
**RF (pos-neg)**	0–9	NA						
	**Mean** **±** **SD (Min-Max)**		***p***	**Adjusted mean** **±** **SEM**		***p***	**Partial correlation with age**	
	**JIA**	**SA**		**JIA**	**SA**		**r2**	***p***
Age (year)	7.6 ± 3.4 (3–14.7)	3.9 ± 4.1 (0.6–12)	0.012					
CRP (mg/ml)	15.3 ± 6.3 (0.3–70.3)	53.4 ± 11.9 (14.6–148.1)	0.0012	5.6 ± 8.3	62.3± 8.0	<0.01	0.42	0.04
Platelets (× 10^6^/ml)	358.3 ± 17.5 (270.0–501.0)	341.6 ± 27.2 (166.0–503.0)	0.877	NA	NA		−0.79	0.0001
WBC (× 10^6^/ml)	8.8 ± 1.1 (4.8–20.4)	14.1 ± 1.5 (6.9–24.4)	0.0024	9.1 ± 1.92	13.7 ± 1.3	0.02	−0.14	0.49

F, female; M, male; JIA, juvenile idiopathic arthritis; SA, septic arthritis; SD, Standard deviation; SEM, Standard error of the mean; ANA, Anti-nuclear antibodies; CCP, cyclic citrullinated peptide; RF, Rheumatoid factor; CRP, C-reactive protein; WBC, white blood cells; r2, partial coefficient correlation controlling for arthritis types; p, p-value; pos, positive; neg, negative.

## Results

### Clinical and Biological Parameters of Children With Arthritis

In total, we included 29 children [*n* = 14 with oligoarticular JIA, *n* = 1 with enthesitis-related arthritis (patient I-03), and *n* = 14 with SA caused by *K. kingae* (*n* = 8), *Staphyloccocus aureus* (*n* = 3; patients C1-05, C1-14, C1-17), *Staphylococcus epidermis* (*n* = 1; C1-16), *Streptococcus pyogenes* (*n* = 1; patient C1-04) or *pneumoniae* (*n* = 1; patient C1-18)] (Table 1). The mean duration between the onset of the occurring inflammatory flare start date and the inclusion were 55.8 ± 63.4 days (min 4 to max 229 days) for JIA, with 50% of the patients included at the first inflammatory flare; and 3.6 ± 2.6 days (min 0 to max 10) for SA. Children with SA were significantly younger than patients with JIA (3.9 ± 4.1 vs. 7.6 ± 3.4), we thus performed multivariate linear regression models, to consider the age difference between the two groups, and confirm or not the age-adjusted significant difference between the two groups. Concerning the biological parameters, significant difference in CRP and blood WBC counts were found between both diseases, and confirmed with age-adjusted values (Table 1). However, a considerable overlap of the cutoff values for CRP and substantial overlapping values for WBC counts were observed.

### Differential Accumulation of T Cells in Patients With JIA Compared With Patients With SA

As previous works showed that WBC counts in PB or SF samples discriminate poorly between JIA and SA (22), we quantified the main immune cell types in both body fluids using flow cytometry (Table 2). Our results confirm that, in PB, the T, B, NK, and granulocytes numbers are not significantly different between the two diseases. In SF, T cell numbers were significantly higher (4-fold) in JIA than in SA, while granulocyte numbers were significantly higher (13-fold) in SA than in JIA. The age-adjusted T cell counts in SF remained significantly different between groups, with however substantial overlapping values.

**Table 2:** Immune cell counts in peripheral blood and synovial fluid samples.

	**Peripheral blood**							
	**Mean** **±** **SD/μl (Min–Max)**			**Adjusted mean** **±** **SEM/μl**			**Partial correlation with age**	
	**JIA**	**SA**	***p***	**JIA**	**SA**	***p***	**r2**	***p***
T-cells	2,461 ± 2,468 (615–11,084)	1,887 ± 1,085 (817–4,445)	0.721	2,792 ± 499	1,533 ± 519	0.219	−0.58	0.002
B cells	761 ± 728 (196–3,076)	680 ± 371 (243–1,376)	0.885	896 ± 141	535 ± 147	0.219	−0.63	< .001
NK cells	427 ± 566 (80–2,419)	329 ± 151 (134–619)	0.885	479 ± 113	273 ± 117	0.382	−0.49	0.001
Granulocytes	3,209 ± 2,043 (1,529–9,463)	4,610 ± 2,352 (1,438–8,987)	0.197	3,079 ± 607	4,749 ± 631	0.181	0.11	0.584
	**Synovial Fluid**							
T cells	1,472 ± 1,015 (573–4,197)	384 ± 308 (36–1,125)	0.001	1,573 ± 203	276 ± 211	0.005	−0.28	0.156
B cells	45 ± 46 (3–131)	46 ± 42 (2–140)	0.885	54 ± 11	36 ± 11	0.416	−0.35	0.071
NK cells	161 ± 209 (22–885)	532 ± 638 (8–2,213)	0.299	149 ± 130	546 ± 135	0.160	−0.21	0.294
Granulocytes	2,110 ± 3,872 (214–15,231)	28,556 ± 42,184 (388–162,512)	0.001	−715 ± 8,010	31,583 ± 8,323	0.066	−0.19	0.323

SD, Standard deviation; SEM, Standard error of the mean; r2, partial coefficient correlation controlling for arthritis types; p, p-value.

### Differences in Monocyte and DC Subsets Discriminate Between JIA and SA

**Figure 1:**
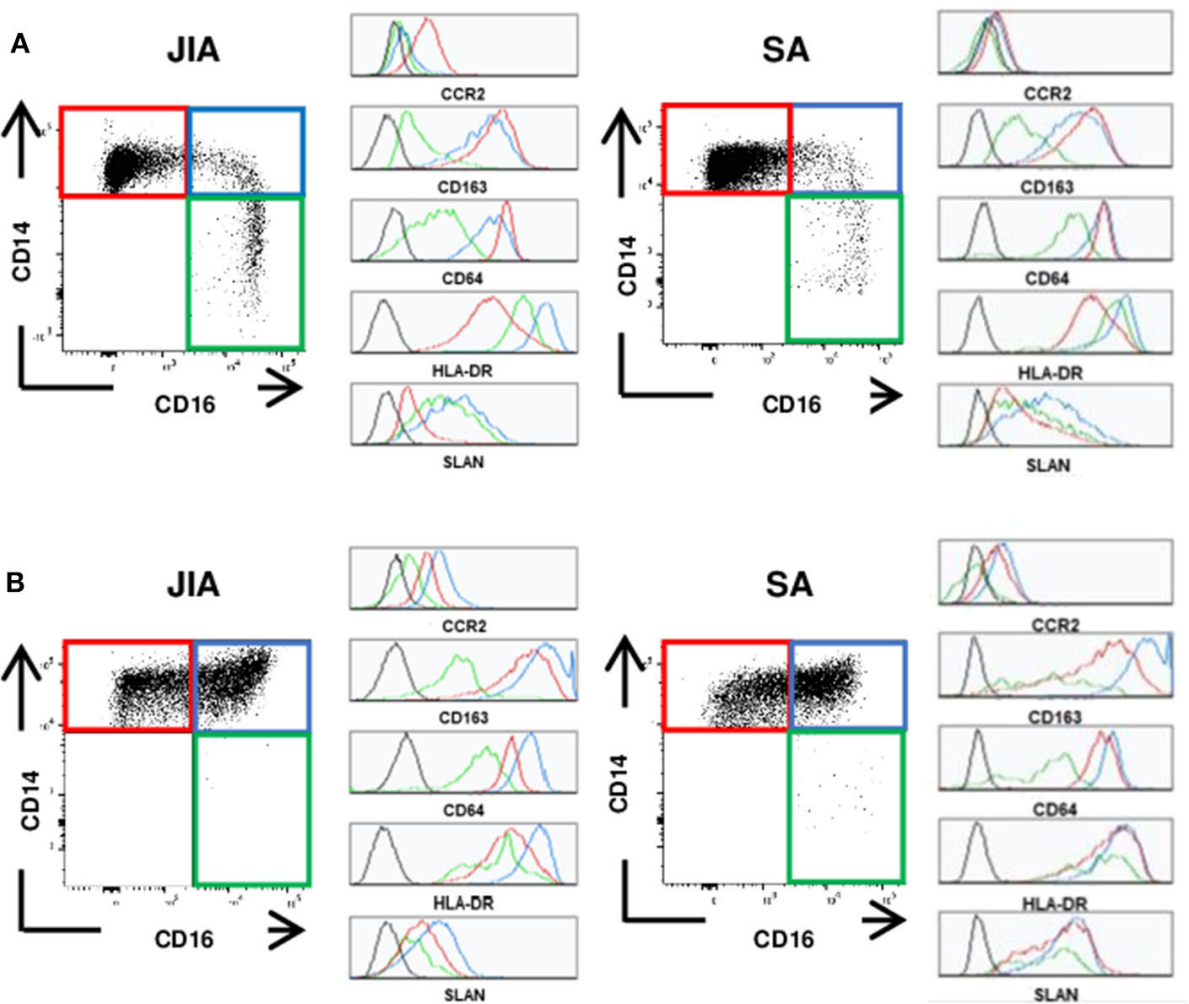
Representative staining of monocyte subsets. Representative dot plots of the classical (CD14^++^CD16^−^, red), intermediate (CD14^++^CD16^+^, blue) and non-classical (CD14^+^CD16^++^, green) monocytes and representative histograms for the various markers (CCR2, CD163, CD64, HLA-DR, and SLAN) on each subset. **(A)** in peripheral blood **(B)** in synovial fluid of patients with JIA (left) or SA (right). Black histograms correspond to negative control MFI.

**Figure 2:**
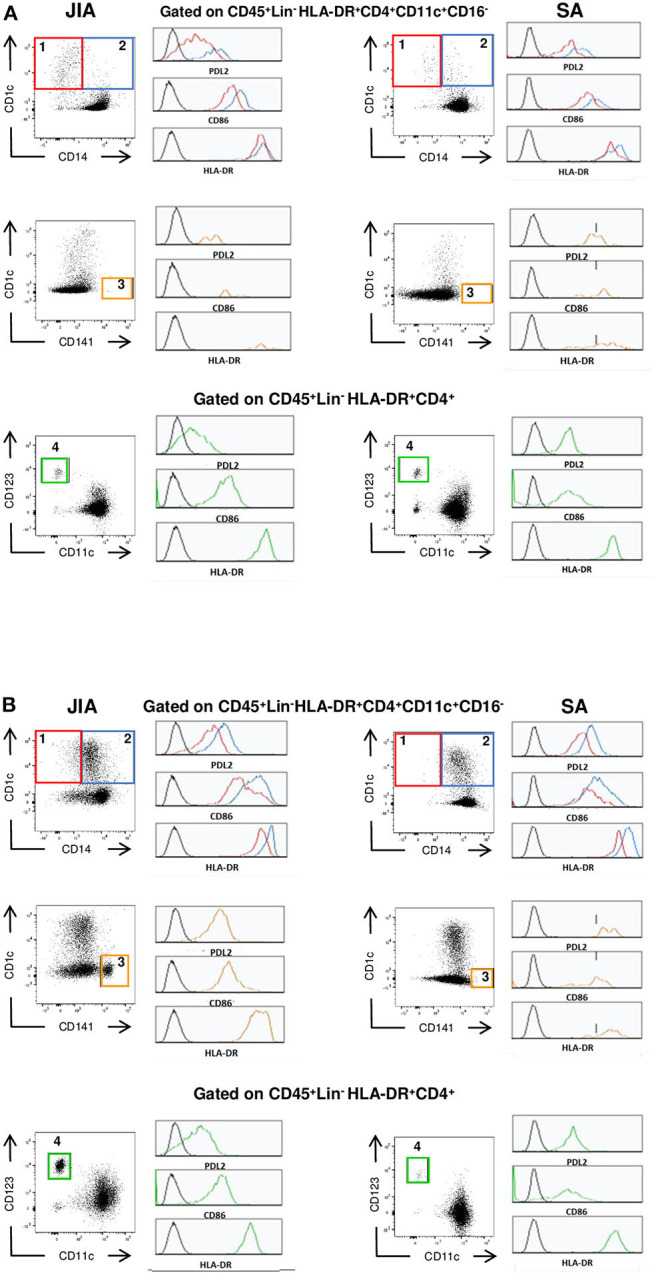
Representative staining of DC subsets. Representative dot plot of the CD1c^+^ cDC (1, red), CD14^+^ InfDCs (2, blue), CD141^+^ cDC (3, orange) and CD123^+^ pDC(4, green) and representative histograms for the various markers (PDL2, CD86, HLA-DR) on each subset **(A)** in peripheral blood and **(B)** in synovial fluid of patients with JIA (left) or SA (right). Black histograms correspond to negative control MFI.

To determine whether innate immune cells could better discriminate between patients with JIA and SA, we immunophenotyped the monocyte and DC subsets in the PB and SF using multi-parametric flow cytometry. All three main monocyte subsets (CD14^++^CD16^−^ classical, CD14^++^CD16^+^ intermediate, and CD14^+^CD16^++^ non-classical) were present in PB samples, whereas non-classical monocytes were barely detectable in SF samples (Figure 1). We could however quantify all four main DC subsets (CD1c^+^ and CD141^+^ cDCs, CD123^+^ pDCs, and CD14^+^ infDCs) in both fluids (Figure 2).

**Figure 3:**
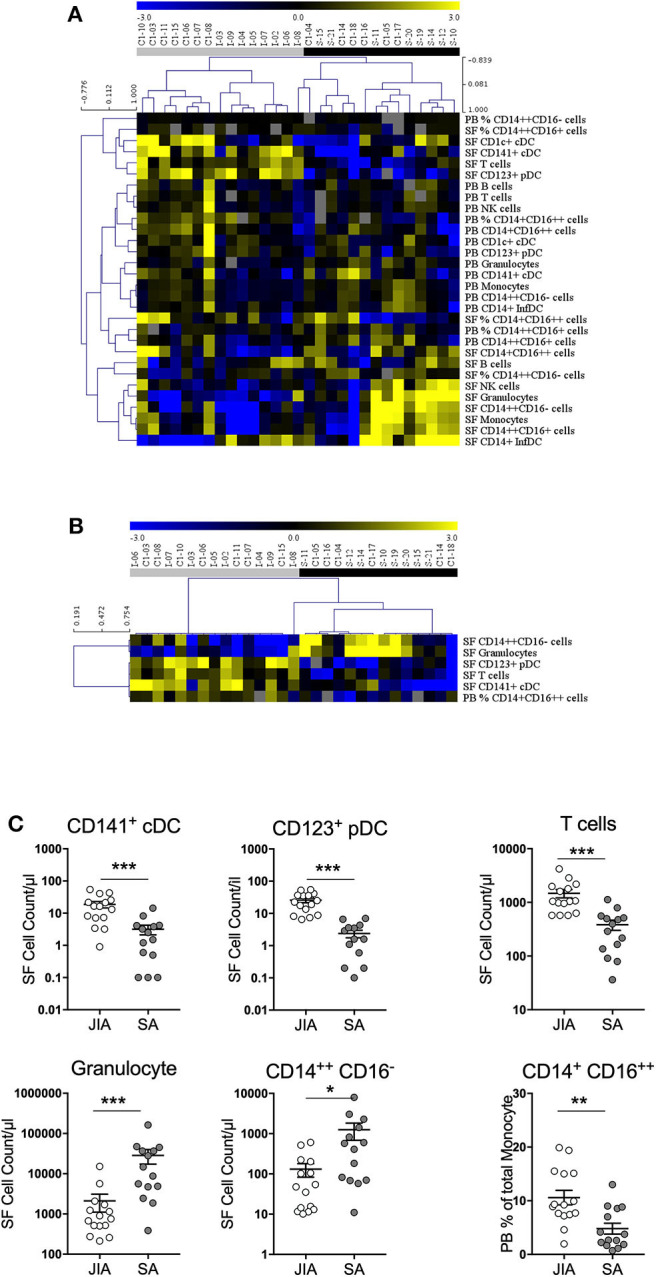
Stratification of patients with JIA and SA based on monocyte proportion and all cell counts. **(A)** Heatmap of monocyte proportion and all cell counts in peripheral blood (PB) and synovial fluid (SF) by multi-parametric flow cytometry. In the color scale, yellow indicates relative higher and blue relative lower cell numbers/proportion. Gray data are undetermined. The dendrogram shows the results of the non-supervised hierarchical clustering analysis; in the heatmap: rows, cell subsets; and columns, individual patients with JIA (gray, *n* = 15) and SA (black, *n* = 14). **(B)** Heatmap showing markers that were significantly differentially expressed between patients with SA and JIA. The dendrogram shows the results of the non-supervised hierarchical clustering analysis using an FDR cut-off of 5%. **(C)** Direct comparison of the 5 differentially expressed cells counts in synovial fluid (SF) and non-classical monocyte proportion in peripheral blood (PB) from JIA (white, *n* = 15) and SA (gray, *n* = 14) patients. Data are represented for all patients and horizontal bars are the mean **±** SEM. *p < 0.05; **p < 0.01; ***p < 0.001; ****p < 0.0001 (two-sided *t* or Mann–Whitney test, according to data distribution after FDR adjustment).

Non-supervised hierarchical clustering analysis of all cell numbers and monocyte proportions (Figure 3A) clearly differentiated samples in two major clades that stratified patients in two groups (JIA and SA) with an exception for one JIA patient (I-08). Analysis using an FDR cut-off of 5% to include only cell counts and monocyte proportions that were differentially represented between JIA and SA samples (Figure 3B) showed, except for the I-08 JIA patient, a clear separation between SA and JIA using only six out of the thirty studied parameters (five in SF and only one in PB). This result suggests a pattern of disease-related changes mainly within joints. Among the five SF parameters, granulocytes and classical monocytes counts were higher in SA than JIA patients and in contrast, the counts of total T cells, CD123^+^ pDCs, and CD141^+^ cDCs were higher in JIA than in SA. In the blood, only the percentage of non-classical monocytes was higher in JIA than SA patients. Overall, these data suggest that monocytes and granulocytes are over-represented in SA, while T cells and DCs are predominant in JIA.

Then, a direct comparison of the discriminating markers was performed. Although CD141^+^ cDCs were barely detectable in PB samples from both groups (Figure 2), we could easily detect and quantify them in SF samples from patients with JIA and their number was significantly higher than in patients with SA (Figure 3C). Similarly, pDCs were significantly higher in SF samples from patients with JIA than with SA with a clear cut-off value of 7 between the two groups. Consistent with previous report in adult forms of arthritis, CD14^+^ infDCs were barely detectable in PB samples from both groups, but represent the major DC subset in SF (Figure 2). However, CD14^+^ infDCs cell count was not different between groups. Altogether, our data demonstrated a significant accumulation of CD141^+^ cDCs and CD123^+^ pDCs in the SF of patients with JIA that was confirmed by comparison of the age-adjusted values (Table 3).

**Table 3:** Proportion, cell counts and mean fluorescence intensities of activation markers on monocytes and DC subsets that discriminate between JIA and SA.

	**Peripheral blood**							
	**Mean** **±** **SD/μl (Min-Max)**			**Adjusted Mean** **±** **SEM/μl**			**Partial corr with age**	
	**JIA**	**SA**	***p*-value**	**JIA**	**SA**	***p*-value**	**r2**	***p*-value**
% CD14^+^CD16^++^ Mono	11 ± 5 (2–20)	5 ± 4 (1–13)	0.008	11 ± 1	4 ± 1	0.006	−0.32	0.091
MFI CD64 CD14^++^CD16^+^	6,515 ± 3,383 (2,770–13,734)	11,029 ± 4,539 (4,555–18,080)	0.031	6,656 ± 1,090	10,866 ± 1,179	0.085	−0.11	0.575
MFI CD64 CD14^+^CD16^++^	620 ± 362 (125–1,165)	1,661 ± 1,161 (568–3,959)	0.026	525 ± 221	1,771 ± 239	0.014	0.10	0.607
MFI SLAN CD14^+^CD16^++^	1,243 ± 772 (264–3,022)	562 ± 334 (131–1,305)	0.031	1,176 ± 163	640 ± 176	0.150	0.20	0.323
MFI CD86 CD1c^+^ cDC	4,018 ± 1,543 (1,827–7,099)	6,197 ± 2,139 (2,543–10,013)	0.028	4,129 ± 529	6,078 ± 551	0.093	−0.20	0.336
MFI PDL2 CD141^+^ cDC	5,520 ± 3,294 (978–10,572)	11,212 ± 3,820 (6,876–18,224)	0.001	5,272 ± 966	11,499 ± 1,045	0.005	−0.02	0.930
	**Synovial fluid**							
% CD14^++^CD16^−^ Mono	23 ± 14 (6–41)	43 ± 22 (13–81)	0.066	20 ± 5	46 ± 5	0.014	0.30	0.115
% CD14^++^CD16^+^ Mono	74 ± 15 (46–92)	56 ± 23 (15–85)	0.060	77 ± 5	53 ± 5	0.033	−0.28	0.144
CD14^++^CD16^−^ Mono	131 ± 188 (10–603)	1,256 ± 2,139 (11–7,971)	0.031	−128 ± 385	1,534 ± 400	0.052	−0.12	0.544
CD141^+^ cDC	19 ± 16 (1–54)	3 ± 4 (0–14)	0.001	20 ± 3	2 ± 3	0.017	−0.10	0.615
CD123^+^ pDC	26 ± 16 (7–54)	2 ± 2 (0–7)	0.001	27 ± 3	1 ± 3	<0.001	−0.35	0.065
MFI CD64 CD14^++^CD16^−^	10,333 ± 4,280 (5,724–20,257)	17,246 ± 6,921 (7,003–35,004)	0.014	10,538 ± 1,584	17,026 ± 1,646	0.064	0.02	0.901
MFI CD64 CD14^++^CD16^+^	17,769 ± 7,885 (10,299–35,053)	28,065 ± 9,342 (17,301–50,314)	0.008	17,492 ± 2,394	28,362 ± 2,487	0.039	0.06	0.750
MFI PDL2 CD141^+^ cDC	8,064 ± 6,184 (1,780–20,214)	22,830 ± 7,438 (14,718–39,379)	0.001	8,298 ± 1,893	22,579 ± 1,967	0.001	−0.15	0.444
MFI PDL2 CD123^+^ pDC	1,496 ± 969 (458–3,183)	2,758 ± 1,257 (1,014–4,662)	0.031	1,570 ± 316	2,673 ± 344	0.140	−0.09	0.641
MFI PDL2 CD14^+^ infDC	4,227 ± 1,935 (2,638–10,386)	9,357 ± 3,466 (4,496–16,379)	0.001	3,679 ± 701	9,944 ± 728	<0.001	0.37	0.052
MFI HLA-DR CD14^+^ infDC	43,795 ± 37,343 (4,785–125,881)	71,214 ± 16,589 (40,691–103,449)	0.066	43,517 ± 8,143	71,511 ± 8,461	0.123	−0.11	0.572
MFI HLA-DR CD123^+^ pDC	17,162 ± 8,005 (6,656–31,243)	27,285 ± 9,616 (9,355–39,606)	0.028	18,064 ± 2,477	26,244 ± 2,693	0.158	−0.30	0.123
MFI CD163 CD14^++^CD16^+^	38,910 ± 17,182 (15,499–80,164)	84,520 ± 45,056 (15,288–169,404)	0.008	40,189 ± 9,331	83,151 ± 9,695	0.038	−0.02	0.913
MFI CD86 CD141^+^ cDC	7,554 ± 4,318 (1,505–17,194)	14,340 ± 7,304 (6,980–30,321)	0.014	6,689 ± 1,572	15,267 ± 1,634	0.014	0.14	0.483
MFI CD86 CD123^+^ pDC	1,558 ± 784 (39–3,021)	3,834 ± 2,975 (479–9,871)	0.034	1,391 ± 597	4,028 ± 648	0.058	0.13	0.520

*r2, partial coefficient correlation controlling for arthritis types; Partial corr with age, Partial correlation with age*.

Comparison of the data on the different monocyte subsets showed that in SF samples, only classical monocytes counts were significantly higher in SA than in JIA (10-fold), whereas in the PB proportion of non-classical monocytes was significantly increase in JIA patients (Figure 3C). The total count of monocytes (sum of all subsets) was higher, although non-significantly, in SA than in JIA (in PB samples: 389 ± 261 in JIA vs. 529 ± 297 in SA, *p* = 0.225; in SF samples: 567 ± 665 in JIA vs. 2,926 ± 4,243 in SA, *p* = 0.101).

### Specific Activation Markers on Monocyte and DC Subsets Discriminate Between SA and JIA

**Figure 4:**
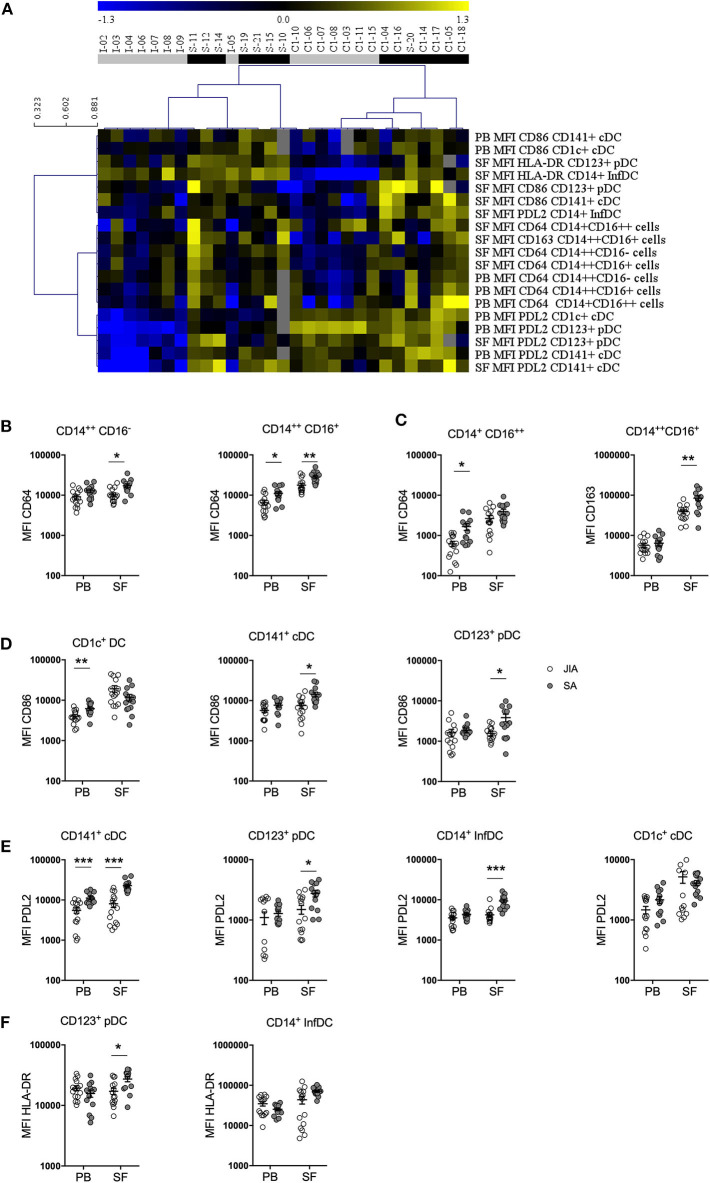
Stratification of patients with JIA and SA based on DC and monocyte activation markers. **(A)** Heatmap showing the mean fluorescence intensities (MFI, rows) of activation markers that were differentially expressed in JIA (gray, *n* = 15) and SA (black, *n* = 14) patients (column). The dendrogram shows the results of the non-supervised hierarchical clustering analysis using an FDR cut-off of 5%. Yellow indicates relative higher expression, blue relative lower expression of the markers and gray undetermined data. Direct comparison of the expression of **(B)** CD64 and **(C)** CD163 in monocyte subsets and **(D)** CD86, **(E)** PDL2, and **(F)** HLA-DR in DC subsets in blood (PB) and synovial fluid (SF) samples of patients with JIA (white, *n* = 15) and SA (gray, *n* = 14); Data are represented for all patients and horizontal bars are mean ± SEM. *p < 0.05; **p < 0.01; ***p < 0.001; ****p < 0.001 (two-sided *t* or Mann–Whitney test, according to data distribution after FDR adjustment).

As monocytes and DCs regulate the T-cell responses against foreign and self-antigens, we analyzed their activation status by monitoring the expression levels of the co-stimulatory HLA-DR and CD86 and of the inhibitory PDL2 receptor in the four DC subsets, and of HLA-DR, CD163, SLAN, CD64, and CCR2 in the three monocyte subsets in PB and SF samples. Non-supervised hierarchical clustering analysis of the MFI data stratified patients in several clades corresponding either to JIA or SA patients with minor exception and similar analysis of the MFI values using an FDR cut-off of 5% (Figure 4A) showed seven clades separating SA and JIA with the exception of one SA patient (patient S-11) and two JIA patients (patients I-05 and C1-10). The separation of the 7 clades are based on eleven SF and eight PB parameters out of the 54 studied parameters. Interestingly, all nineteen activation markers that discriminated both diseases were more strongly expressed in SA than in JIA, and included seven monocyte and twelve DC activation markers. Expression of CD64 and CD163 on monocytes and PDL2, CD86, and HLA-DR on DC discriminate both type of arthritis.

Direct comparison of the differences in expression levels of activation markers in the monocyte subsets showed that CD64 expression on the three monocyte subsets in both fluids (Figure 4B) as well as CD163 expression in intermediate monocyte in the SF (Figure 4C) were higher in SA than in JIA. Statistical analyses confirmed the majority of these results and also revealed that SLAN expression were significantly increased in the non-classical monocyte subset of the PB in JIA compared with SA (Table 3). In our study CCR2 were similarly expressed in both JIA and SA groups (data not shown). Overall, expression of CD163, SLAN, and CD64 in monocyte subsets was different between JIA and SA; however, after age-adjustment, only CD64 and CD163 MFI remained significantly higher in SA than in JIA. CD64 was over-expressed on non-classical monocytes in PB and classical and intermediate monocyte in SF (Table 3).

Among the four CD86 selected parameters on the heatmap, analysis of the differences in expression levels of activation markers on DC subsets revealed that CD86 expression was significantly increased in CD1c^+^ from PB and in CD141^+^ cDCs and CD123^+^ pDCs from SF samples of patients with SA (Figure 4D). Among the six PDL2 selected parameters on the heatmap, PDL2 expression level was significantly increased in CD141^+^ cDC of PB and on CD141^+^ cDC, CD123^+^ pDC, and CD14^+^ InfDC from SF of patients with SA compared with JIA (Figure 4E). HLA-DR expression level in SF was higher in CD123^+^pDCs and CD14^+^ infDCs in patients with SA compared with JIA (Figure 4F). All these differences were confirmed on age-adjusted values except for CD86 expression on PB CD1c^+^ cDC and HLA-DR and PDL-2 expression on SF CD123^+^ pDC (Table 3).

### CD123^+^ pDC and CD141^+^ cDC Counts and PDL2 Expression in DCs Discriminate Between JIA and SA

**Figure 5:**
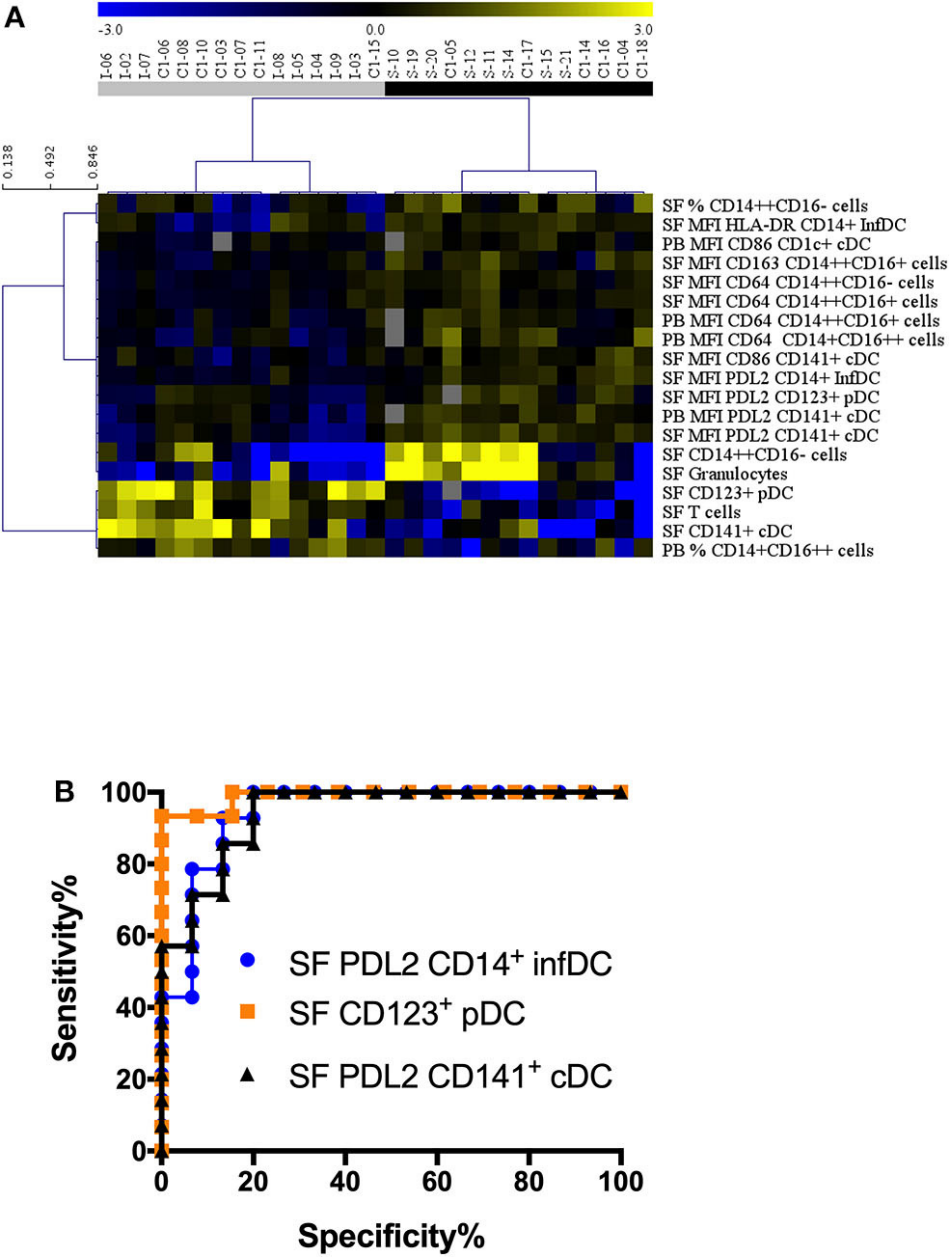
Stratification of patients with JIA and SA based on all parameters. **(A)** Heatmap showing the markers (markers, rows) that were differentially represented in JIA (gray, *n* = 15) and SA (black, *n* = 14) patients (column). The dendrogram shows the results of the non-supervised hierarchical clustering analysis using an FDR cut-off of 5%. Yellow indicates relative higher expression, blue relative lower expression of the markers. **(B)** Receiver operating curves (ROC) for markers with AUC values > 0.94.

Non-supervised hierarchical clustering using an FDR cut-off of 5% on all cell counts/proportions and MFI cytometry parameters (*n* = 84) showed clear separations between JIA and SA using fourteen SF and five PB parameters (Figure 5A). Among all the parameters that were significantly different between diseases after age-adjustment, seven biomarkers displayed an AUC value >0.85 in the ROC curve analysis, suggesting that they could be used to discriminate between JIA and SA (Table 4). In PB, only PDL2 expression on CD141^+^ cDCs could be retained. The six parameters in the SF are: PDL2 expression on CD14^+^ InfDC and CD141^+^ cDCs; the number of CD123^+^ pDCs and CD141^+^ cDCs, and granulocytes and T cell counts. The ROC curves of the three highest AUC are shown in Figure 5B. These data suggest that monitoring DC subsets in SF samples and/or PDL2 expression in DCs from PB or SF samples may be a convenient and rapid strategy to discriminate between both diseases at diagnosis.

**Table 4:** Parameters highly significantly different between diseases after age-adjustment, with AUC value >0.85 in the ROC curve analysis.

	**Mean** **±** **SD/μl (Min-Max)**							
	**Peripheral blood**							
	**JIA**	**SA**	**p**	**Youden cutt-off**	**Specificity**	**Sensitivity**	**AUC**	***p***
MFI PDL2 CD141^+^ cDC	5,520 ± 3,294 (978–10,572)	11,212 ± 3,820 (6,876–18,224)	0.001	6,876	0.6000	1.0000	0.8667	0.0004
	**Synovial fluid**							
CD141^+^ cDC	19 ± 16 (1–54)	3 ± 4 (0–14)	0.001	4.22	0.8000	0.8571	0.8762	<0.0001
Granulocytes	2,110 ± 3,872 (214–15,231)	28,556 ± 42,184 (388–162,512)	0.001	1,868	0.8000	0.9286	0.8810	<0.0001
T cells	1,472 ± 1,015 (573–4,197)	384 ± 308 (36–1,125)	0.001	558	1.0000	0.8571	0.9381	<0.0001
MFI PDL2 CD141^+^ cDC	8,064 ± 6,184 (1,780–20,214)	22,830 ± 7,438 (14,718–39,379)	0.001	14,718	0.8000	1.0000	0.9429	<0.0001
MFI PDL2 CD14^+^ infDC	4,227 ± 1,935 (2,638–10,386)	9,357 ± 3,466 (4,496–16,379)	0.001	4,495	0.8000	1.0000	0.9429	<0.0001
CD123^+^ pDC	26 ± 16 (7–54)	2 ± 2 (0–7)	0.001	7	0.9333	1.0000	0.9905	<0.0001

## Discussion

Due to ethical and technical reasons, heterogeneity of DC and monocyte subsets are poorly described in human tissue and biological fluid other than PB (25). Recruitment of specific DC and/or monocytes in inflamed tissues increase this heterogeneity and their activation status could play a key role in the pathophysiology of the disease promoting T helper cell polarization depending on the environment. Using high-dimensional protein and RNA-single-cell analyses, Ginhoux's team recently analyzed human-circulating DC and monocyte subsets. They revealed high heterogeneity of myeloid cells with expansion of a specific cDC2 subset characterized by expression of CD163 and CD14 and correlating with systemic lupus erythematosus (SLE) progression (26). Our present study revealed heterogeneity of DC and monocytes subsets in the SF from SA and JIA patients and provided a unique opportunity to identify specific DC and monocytes subsets with known functional specialization that could be associated with a pathophysiological status in inflammatory settings. The markers for DC and monocytes subsets were however analyzed in two different 17-parameters panels and did not allowed us to discriminate monocytic markers such as CD163 on DC subsets and particularly on CD14^+^ infDC. Combining all the studied markers in a single panel will be needed to further characterized more precisely the delineation between inflammatory monocytes and DCs.

To discriminate between the two pathophysiological processes, we first quantified WBC, T, B, NK cells and granulocytes in PB and SF from SA and JIA. Our results showed that, in SF samples, the mean number of granulocytes was 13-fold higher in patients with SA than with JIA, and the mean count of T cells was 4-fold higher in patients with JIA than with SA.

Massive granulocytes recruitment to the inflamed site is one of the first step of the immune response. In our study, comparing patients with low and intermediate SF WBC counts (5 to 75 × 10^3^/μl) the multivariate linear regression model analysis with age-adjusted values tend to confirmed the significant difference in granulocyte counts between SA and JIA. Significant T cell increase was observed in SF from JIA, confirming previous studies showing that activated T cells accumulate in the synovial membrane, forming clusters around antigen-presenting cells (e.g., DCs) (27, 28), and might trigger the autoreactive immune response. However, both T and granulocytes cell counts in the SF showed substantial overlapping values between both groups of patients that limits their use as relevant biomarkers. Therefore, we immunophenotyped the main monocyte and DC subsets in PB and SF analyzing 76 parameters (percentage, counts and activation status). In the SF, we found that percentage of classical monocyte subset, as well as absolute counts, were significantly higher in SA compared with JIA, and both results were confirmed by the age-adjusted analyses. In contrast significant increase of the percentage of intermediate monocyte subsets was found in JIA compared with SA and this result was also confirmed by the age-adjusted analyses. In PB, the percentage of non-classical monocyte subsets was significantly higher in JIA compared with SA and this result was confirmed following age-adjusted analyses.

The increased proportion and absolute numbers of both intermediate and non-classical monocytes was previously observed in PB of children with sepsis (16, 29). Moreover, CD16^+^ monocytes are increased in various infectious and inflammatory diseases (e.g., AIDS, asthma, chronic infections, notably sepsis) (29), as well as in metabolic and autoimmune disorders (e.g., obesity, multiple sclerosis, SLE and rheumatoid arthritis) (15). In addition, in PB of SA patients, the CD14^+^CD16^++^ subset has a reduced HLA-DR expression, although non-significant, similarly to what was previously described in sepsis (16, 29, 30). We also showed that it is associated with a significant higher CD64^+^ expression than in JIA. Consistent with our findings, a higher HLA-DR expression in synovial intermediate monocytes was also observed in patients with enthesis's-related JIA (18). CD64 is a high-affinity FcγRI receptor with restricted isotype-specificity that is expressed on macrophages, monocytes, neutrophils, and eosinophils. A previous study suggested that CD64 expression on monocytes could be used to assess the IFN type I status in patients with systemic lupus erythematosus (SLE) (31), and could be included in the diagnostic work-up of sepsis and infection in adults (32–34) and in children (35–37). Consistent with the study by Hofer et al. that described SLAN as a marker of CD16^+^ monocyte subsets in PB (38), we found that SLAN is mainly expressed in blood CD16^+^ monocytes, and significantly increased in the PB of JIA. Interestingly, in SF samples, SLAN expression is comparable in all monocyte subsets and between diseases. The analysis after adjustment for age, confirmed the high expression of CD163 on SF intermediate monocytes and CD64 on PB non-classical monocytes and SF classical and intermediate subsets in SA patients.

In blood, we did not observe any significant difference in the count of the four main DC subsets between both forms of juvenile arthritis, suggesting that the distribution of DC subsets in the blood is not informative. This is consistent with the recent study by Throm et al. showing no difference in pDC and mDCs distribution between treatment-naive and remission patients with JIA and with matched controls using mass cytometry (39). In contrast, in the SF, we observed a very significant accumulation of CD141^+^ cDCs and CD123^+^pDCs in JIA patients with a clear cut-off value of 7 for pDCs and these results were confirmed after adjustment for age. Such accumulation of both types of DCs was previously reported in SF from JIA patients when comparing with PB (7). Maturation of blood and SF DCs was evidenced by the high expression of HLA-class II and CD86 co-stimulatory molecules in the four DC subsets in both diseases. A higher expression of HLA-DR molecules was observed on CD123^+^ pDC and CD14^+^ infDCs in SA SF samples compared with JIA. Moreover, we observed a significant up-regulation of CD86, in PB CD1c^+^ cDCs and in SF CD141^+^ cDCs and CD123 pDC from SA patients. Interestingly a potent activation of PB and SF CD14^+^ infDCs in both diseases were observed with high numbers of this particular subset in the SF from SA patient. Blood CD141^+^ DCs excel at cross-presentation of cellular antigens, immune complexes and antigens specifically targeted to late endosomes. This DC subset can polarize CD4^+^ T cells toward the Th1 phenotype, particularly after TLR3 binding, and induces Th2 polarization more potently than CD1c^+^ cDCs. A recent study reported that in the inflamed synovial joint of patients with RA, CD141^+^ cDCs are significantly enriched, can activate CD4^+^ and CD8^+^ T cells, and thus contribute to the synovial joint inflammation (40). Conversely, pDCs, in which MHC class II and co-stimulatory molecules are expressed at lower level, are less efficient at priming T cells (41). In addition, pDCs can induce tolerogenic immune responses by inducing regulatory T cells (42–45), and their increase is associated with RA remission (46). Tissue infiltration by pDCs has been however, described in skin lesions of patients with SLE, psoriasis, and systemic sclerosis (47, 48), in the salivary glands of patients with Sjörgren's disease (49), and in muscle and skin of patients with juvenile dermatomyositis (50). In these autoimmune diseases, CD123^+^ pDCs are the major source of type I IFN and are implicated in inflammation initiation and in the transition to chronic disease (25, 51). Occasional RA onset during type I IFN treatment was reported (52) and cells with phenotypic and functional characteristics of pDCs were found to infiltrate the inflamed synovial tissue in adult RA (53)- (54). Recently pDC heterogeneity had been described with the discovery of AS DCs (positive for *AXL, SIGLEC1* and *SIGLEC6* antigen) within the traditionnally defined pDC population (55) and this heterogeneity might explain the various functional properties previously assigned to pDCs.

Finally, we found that PDL2 expression was significantly increased in several DC subsets isolated from SF and PB samples of patients with SA compared with JIA. Although it was initially thought that PDL2 expression was restricted to antigen-presenting cells, such as macrophages and DCs (56), recent findings have shown that its expression can be induced in many immune and non-immune cell types, depending on the micro-environmental stimuli (57–60). Particularly, PDL2 expression is increased in circulating monocytes and CD4^+^ T lymphocytes in patients with septic shock (61). PDL2 expression is regulated mainly through the NF-κB and signal transducer and activator of transcription-6 (STAT-6) pathways (61, 62). The most potent PDL2 inducers are Th2 cytokines, particularly IL-4. The increased PDL2 expression on DC subsets in SA suggests a feed-back mechanism of T-cell activation. Indeed, PDL2/PD-1 interaction results in the suppression of TCR-induced PI3K/AKT activation and the subsequent reduction of T-cell survival, cytokine production, and proliferation. Overall, these data show that in PB and SF samples of patients with JIA, the expression of the inhibitory PDL2 molecule is decreased, compared with patients with SA.

Infectious and autoimmune diseases have often been associated with altered monocyte or DC numbers, suggesting their pathogenic role in these pathologies. Our results indicate the involvement of monocytes as the first line of defense against pathogens in SA, while DC, and particularly the pDC and CD141^+^ cDC subsets, seem to play a critical role in JIA through the polarization of the T-cell immune response in JIA. We demonstrated, a differential expression of HLA-DR, CD86and PDL2 on DC subsets and CD64 and CD163 on monocyte subsets that reflect various states of activation of both monocytes and DC subsets in the PB or the SF from JIA and SA. The functional consequences of the differential activation of monocytes and DCs in these diseases need to be further investigated. It is important to note that oligoarticular JIA is a chronic disease with flare mimicking acute arthritis. The delay between disease onset and inclusion of the patients in our study is therefore longer for JIA than SA patients. However, the inclusion of the JIA patients was performed mainly in the first month of the onset of the clinical signs of arthritis. Nevertheless, the observed accumulation of pDC and CD141^+^ cDC in SF from JIA patients might be the consequences of the chronic inflammation. These biomarkers would thus be not helpful to discriminate others forms of JIA. To answer this specific question, which is out of the scope of our manuscript, inclusion of patients with other forms of JIA (e.g., psoriatic arthritis and rheumatoid factor positive arthritis) would be needed.

In conclusion, our study contributes to the identification of specific subsets of monocytes and DC potentially implicated in JIA and SA physiopathology and shows a specific accumulation of CD141^+^ cDCs and CD123^+^ pDCs in the SF from JIA patient. The differential diagnosis between SA and JIA is a matter of emergency for proper care in pediatric consultation and despite distinct etiology it remains difficult because of similar sign and symptoms. A recent study from our team identified a 3-miRNA signature in SF (miR-146a-5p, miR-155, and miR-6764-5p) as a highly sensitive and specific potential diagnostic biomarker of JIA, as compared with SA (63). Similar to the present cell-based study, the miRNome-based study of Nziza et al. also showed that SF better discriminate JIA and SA than serum. Both studies have been performed on different cohort but with similar numbers of patients, and only cell surface markers were analyzed on fresh samples. Because joint puncture is part of the clinical management of both diseases, using quantification of any biomarkers from the SF seems to be easily and rapidly feasible in clinical routine. Validation studies on larger independent patient cohort will have to be conducted to determine the predictive power of the identified candidate biomarkers.

## Data Availability Statement

The raw data supporting the conclusions of this article will be made available by the authors, without undue reservation.

## Ethics Statement

The studies involving human participants were reviewed and approved by Comité de Protection des Personnes Sud Méditerranée I ref 2014-A01561-46. Written informed consent to participate in this study was provided by the participants' legal guardian/next of kin.

## Author Contributions

MC performed the phenotypic experiments and analyses, discussed results, and contributed to the study design. ED-L performed experiments and analyses. AC, PM, MD, and EJ recruited patients and discussed the results. HC and MK performed study monitoring and discussed the results. NN, ID-R, FA, and PL-P discussed the results and contributed to the study design. TM performed the statistical analyses and discussed the results. PL-P, FA, and EJ conceived the study, discussed the results, and wrote the manuscript. All authors contributed to the article and approved the submitted version.

## Conflict of Interest

The authors declare that the research was conducted in the absence of any commercial or financial relationships that could be construed as a potential conflict of interest.
