# Supplementary figures and images for: Differential Accumulation and Activation of Monocyte and Dendritic Cell Subsets in Inflamed Synovial Fluid Discriminates Between Juvenile Idiopathic Arthritis and Septic Arthritis
Source: Front Immunol. 2020 Jul 31;11:1716. doi: 10.3389/fimmu.2020.01716 (PMC7411147; doi:10.3389/fimmu.2020.01716)

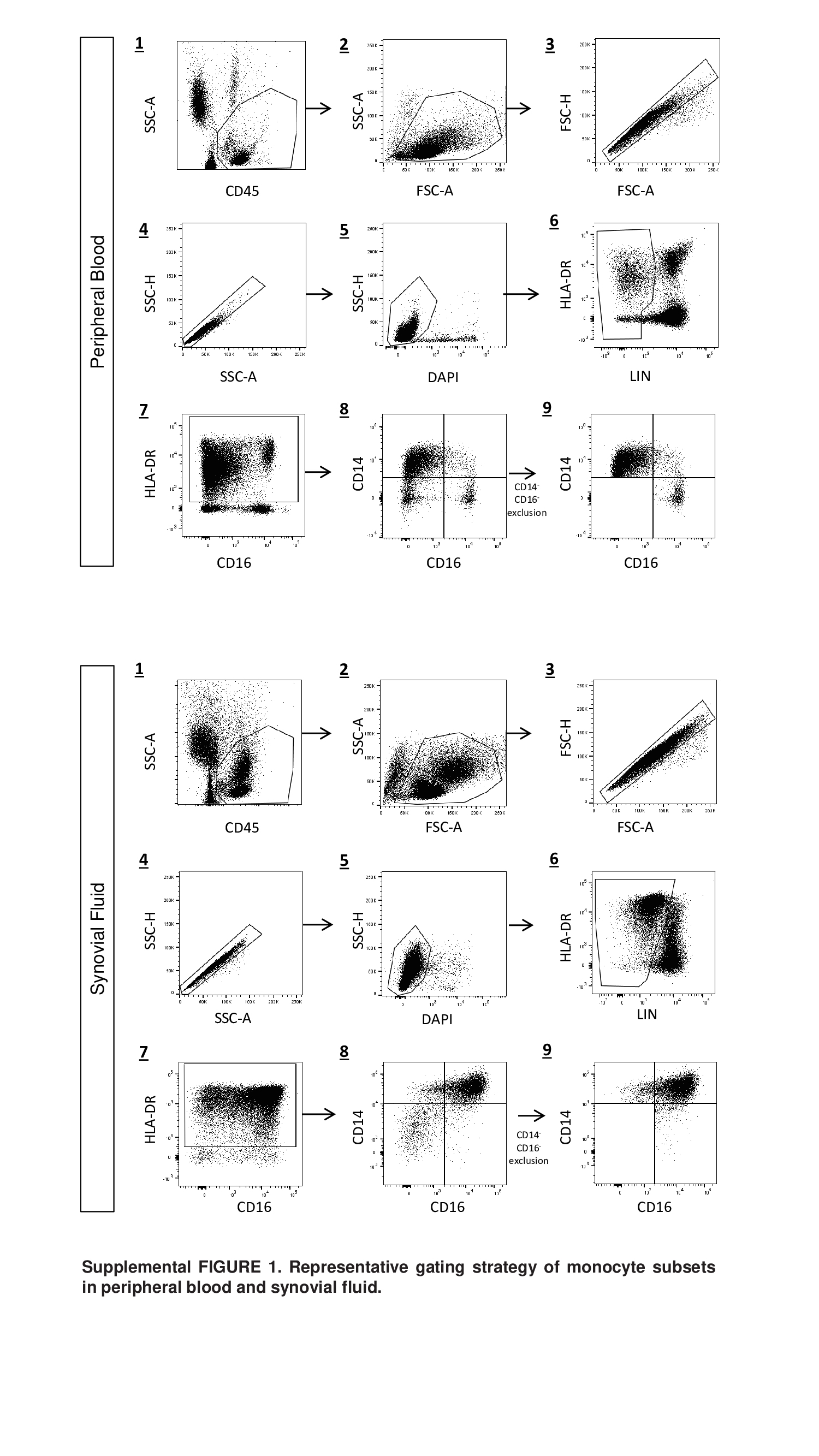

Supplement: Supplementary file 1 [file Image_1.JPEG]

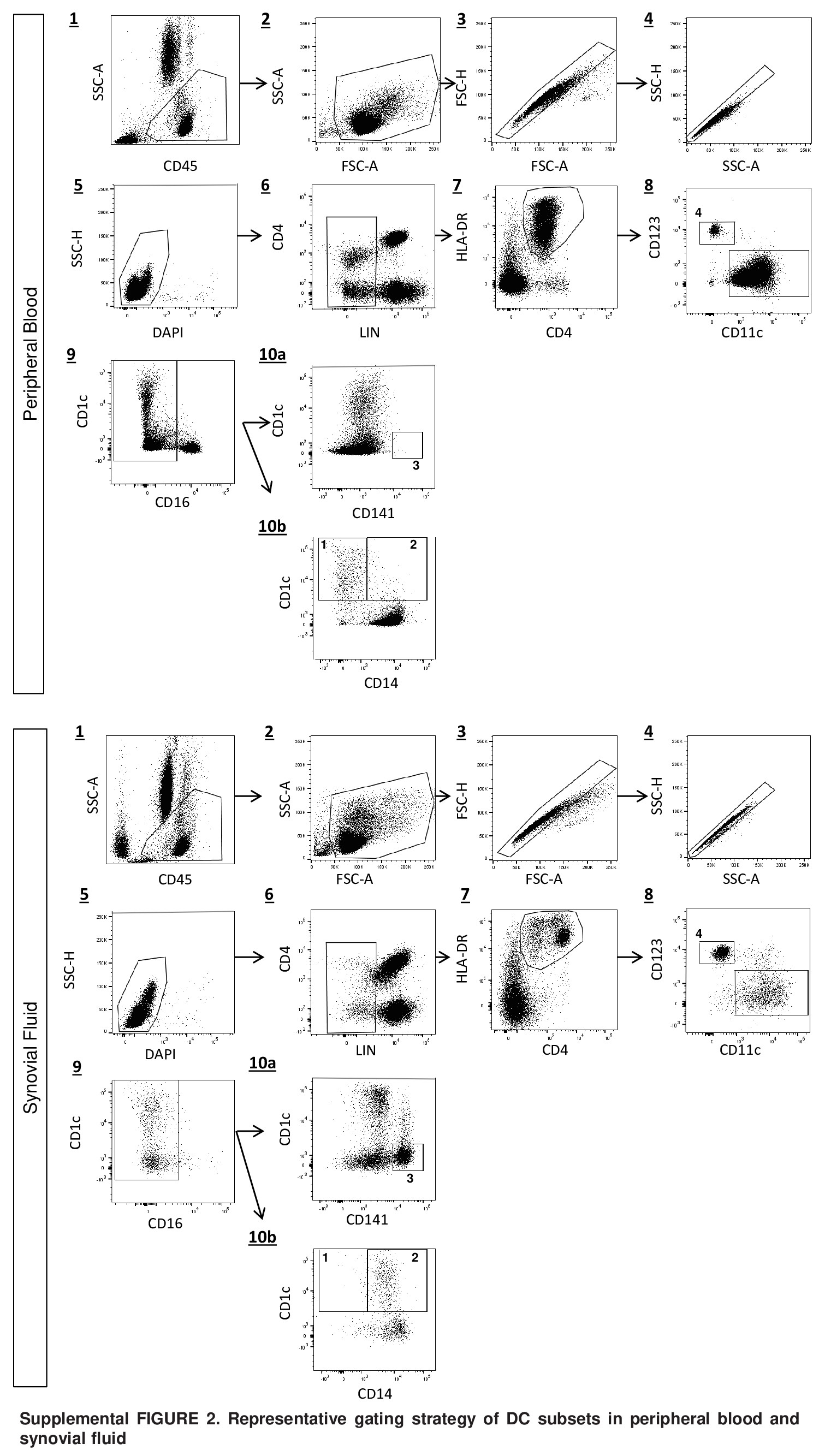

Supplement: Supplementary file 2 [file Image_2.JPEG]
